# Epstein-Barr Virus and Human Cancer

**DOI:** 10.1038/sj.bjc.6600427

**Published:** 2002-07-15

**Authors:** Lawrence Young

**Affiliations:** Cancer Research UK Institute for Cancer Studies, The University of Birmingham, Birmingham, UK

## Abstract

*British Journal of Cancer* (2002) **87**, 28–30. doi:10.1038/sj.bjc.6600427 www.bjcancer.com

© 2002 Cancer Research UK

## 

“Things are seldom what they seem, skim milk masquerades as cream!”

Duet – Little Buttercup and Captain, Act II, *HMS Pinafore*

The study of virus-associated human malignancy has provided many important insights into the pathogenesis of cancer as well as facilitating the development and testing of novel therapeutic strategies relevant to the treatment of common cancers. Epstein-Barr virus or EBV, as the first oncogenic human virus to be identified, has been at the vanguard of these studies and even 40 years after its discovery the virus continues to fascinate and surprise as it yields up its secrets. The study of EBV intersects many disciplines including cell biology, immunology, molecular biology and pathology and this provides an important advantage in tackling the complexity of the tumorigenic process. The virus also provides a useful paradigm for the benefits and challenges associated with the impact of having a complete DNA sequence on our understanding of human disease. The full DNA sequence of the EBV genome, some 172 kilobases, was published in 1984 thus we are almost 20 years into the post-genomic era but the ‘big questions’ relating to such areas as the nature of virus persistence and replication *in vivo* and the precise contribution of the virus to the oncogenic process are only just beginning to be unravelled. This is a salutary lesson that highlights the need for restraint in propounding the immediate benefits of the human genome sequence to the elucidation of disease processes and the development of new treatments. Nevertheless EBV has come a long way and this slender volume edited by Kenzo Takada nicely highlights the current state of the field and sets the scene for the challenges ahead.

‘Epstein-Barr Virus and Human Disease’ is a selective collection of reviews from experts in Japan and the United States covering a range of topics. While considering detailed aspects of EBV biology such as virus replication and the function of EBV-encoded glycoproteins, some key fundamental features of the virus like the function of the latent membrane proteins (LMP1 and LMP2) or the latest theories on the nature and site of EBV persistence are not covered. This reflects the interests of the various authors and with nine of the 14 chapters being provided by Japanese researchers the volume is naturally biased towards certain topics at the expense of others. This is best exemplified in Section II which covers EBV-associated malignancies and provides two chapters on those tumours which are of particularly interest and importance in Japan (namely gastric adenocarcinoma and nasal lymphoma) but relatively rare in the West. The other review on AIDS lymphoma in relation to both EBV and HHV8 seems a little out of place and some consideration of the evidence and recent work implicating EBV in the aetiology of Hodgkin's disease or nasopharyngeal carcinoma (NPC) may have been more appropriate. These minor deficiencies are more than compensated by the editor's own contribution and that of Jeff Sample's group describing their studies using the Burkitt lymphoma (BL) cell line, Akata, to examine the role of EBV in the pathogenesis of BL. Takada was the first to describe the ability of EBV-positive Akata BL cells to lose the virus in culture with consequent loss of the transformed phenotype and his chapter summarises these studies. This system provides a valuable tool for dissecting the role of individual EBV-encoded latent genes in the oncogenic process and for generating recombinant forms of the virus. The use of this approach to examine the impact of EBV infection on cell growth and on c-*myc* expression, a central player in the pathogenesis of BL, is reviewed by Ruf *et al*. Another important contribution from Takada's group has been the use of recombinant drug-resistant forms of EBV to infect epithelial cells and thus Imai *et al* provide a superb review of this area highlighting the association of the virus with NPC and other carcinomas and examining over 20 years' worth of *in vitro* studies attempting to elucidate the nature of the virus-epithelial cell interaction.

The volume also contains a section on the molecular mechanisms of maintenance and disruption of virus latency. Two reviews deal with the factors governing the extrachromosomal maintenance and replication of the circular (episomal) EBV genome. Here the interaction of the EBNA1 protein with the latent viral replication origin, *oriP*, is described and the possible role of cellular licensing factors in controlling replication of the EBV episome is considered. Two chapters cover the other aspect of virus replication, that is the activation of the EBV lytic cycle which results in the production of progeny virus. Tsurimi examines the role of various viral proteins in this complex process and provides a model for the EBV replication fork whereas Fujiwara describes his work on the possible influence of the EBNA2 protein on lytic cycle induction. Lindsey Hutt-Fletcher's chapter on the two major EBV glycoprotein complexes is an important reminder of the critical functions of these membrane proteins in relation to EBV replication and tropism and how these might impact on EBV pathogenesis. The final section of the book deals with animal models and therapeutic approaches. Fred Wang reviews the EBV-like herpesviruses infecting Old World primates and describes the similarities, at both the molecular and biological level, of these so-called lymphocryptoviruses (LCVs). Rhesus LCV is a particularly good model for EBV in terms of tumour association, *in vitro* B cell transformation and the natural history of infection and thus provides a valuable experimental model to study key aspects of EBV pathogenesis such as the role of latent genes in acute and persistent infection, virus immune evasion strategies and virus-associated tumorigenesis. Finally, Clio Rooney and co-workers consider the use of EBV-specific cytotoxic T lymphocytes for the treatment of virus-associated tumours. This group has pioneered this form of adoptive immunotherapy for the treatment of EBV-induced lymphoproliferative disease and the chapter reviews their experience with a comprehensive assessment of the ‘pros and cons’ of this therapy. The possibility of using this approach for the treatment of other EBV-associated tumours such as Hodgkin's disease is also discussed.

‘Things are seldom what they seem’ when it comes to studying virus-associated human tumours. There are many challenges and paradoxes. For instance, EBV is probably one of the most common viral infections throughout the world but only a small proportion of infected individuals develop tumours and these differ dependent on where you reside. This highlights the complexity of oncogenesis and the fact that whilst EBV may be essential in this process other factors working in concert with virus infection are required to fully transform a normal cell. For many years EBV-induced transformation of B lymphocytes in culture was considered to be a model for virus-associated cancers until it was discovered that EBV adopts distinct patterns of latent gene expression in different tumours and that in certain situations such as BL the virus-encoded proteins considered to have oncogenic activity are not expressed! These are important points for post-graduate students and post-doctoral research fellows to appreciate which may not be clear from a reading of this volume. However, the book provides a useful but selective snap shot of the current state of play contrasting our detailed knowledge of certain molecular aspects of EBV biology with almost complete ignorance of fundamentally important processes such as the role of EBV in the pathogenesis of NPC or the contribution of epithelial cell infection to virus persistence and replication. The book also highlights the value of new approaches, particularly the ability to generate EBV recombinants, as well as illustrating how new treatments can be developed without a full understanding of the disease process. There's much still to do and, with an increasing number of viruses being implicated in the development of human cancer, EBV stands as a paradigm for both tumour virology and for the challenges of the post-genomic era.

